# *In Vitro* and *In Vivo* Antigen Presentation and Diagnosis Development of Recombinant Overlapping Peptides Corresponding to *Mtb* ESAT-6/CFP-10

**DOI:** 10.3389/fimmu.2022.872676

**Published:** 2022-06-16

**Authors:** Qing Zhang, Xiong Lu, Liang Gao, Siyu Tao, Yinghua Ge, Daocheng Cui, Renying Zhu, Wenshu Lu, Jian Wang, Shisong Jiang

**Affiliations:** ^1^ College of Veterinary Medicine, Faculty of Animal Science, Southwest University, Chongqing, China; ^2^ R & D Department, Oxford Vacmedix (Changzhou) Co. Ltd., Changzhou, China; ^3^ Department of Tuberculosis, Changzhou Third People’s Hospital, Changzhou, China; ^4^ Department of Clinical Laboratory, Dehong Prefectural Hospital, Dehong Prefecture, China; ^5^ R & D Department, Shanghai JW Inflinhix Co. Ltd., Shanghai, China; ^6^ Department of Oncology, University of Oxford, Oxford, United Kingdom

**Keywords:** recombinant overlapping peptides, mycobacteria tuberculosis, Bovine TB, antigen presentation, immune responses, diagnostic

## Abstract

Cellular immunity in *Mycobacteria tuberculosis* (*Mtb*) infection is important for the pathogenesis and final clearance of intracellular *Mtb* infection. In addition, it is valuable for the diagnosis of tuberculosis. In this pioneering work, we tested *in vitro* and *in vivo* antigen presentation and diagnostic application of a recombinant overlapping peptide-protein derived from two *Mtb* RD1 antigens ESAT-6 and CFP-10 (ROP-TB). The overlapping peptide sequence of ROP-TB is cleaved by the cathepsin S enzyme and covers the entire length of the two proteins. ROP-TB can be expressed and purified from *E. coli*. Once taken in by antigen-presenting cells, ROP-TB can be cleaved into a peptide pool by cathepsin S within the cells. We found that in dendritic cells, ROP-TB can be processed in 6 hours of co-culture, while the ESAT-6/CFP-10 fusion protein remained in the endosomal compartment. In *Mtb*-infected mice, ROP-TB stimulated stronger specific T cell responses than pooled synthetic peptides derived from ESAT-6 and CFP-10. With regard to the presentation of *in vivo* antigens, in a guinea pig model infected with *Mtb*, ROP-TB induced delayed type hypersensitivity (DTH) responses comparable to those of the tuberculin purified protein derivative (PPD) and ESAT-6/CFP-10 fusion protein. In *Mycobacterium bovis (Bovine TB)*-infected cattle, ROP-TB elicited DTH responses. Finally, in *Mtb* infected patients, ROP-TB stimulated cellular immune responses in majority of patients (16/18) of different HLA phenotypes while a single peptide derived from the same proteins did not elicit the immune responses in all patients. In summary, *in vitro* and *in vivo* data suggest that ROP-TB stimulates a strong cellular immune response irrespective of HLA phenotypes and is therefore suitable for use *in vitro* and *in vivo* diagnostics.

## Introduction

Cell-mediated immunity requires antigen presentation by class I or II MHC molecules on the surface of antigen-presenting cells (APC). CD8+ T cells typically recognize epitopes presented with MHC class I derived from endogenous antigen degradation in the cytoplasm of any nucleic cell, including APCs (Class I pathway). In contrast, exogenous antigens are processed in the endosome/lysosome in APC into peptides which can be presented by MHC class II (only expressed in APCs) to the cell surface and stimulate CD4+ T cells (Class II pathway). In some situations, exogenous antigens can be cross-presented in APCs to prime CD8+ T cells (Cross-presentation). However, the efficiency of antigen cross-presentation is low (1–3).

Cellular immunity is very important in the pathogenesis and elimination of *Mtb* infection. Furthermore, since the antibody is not suitable for the diagnosis (4–6), cellular immunity is a useful surrogate diagnostic marker for *Mtb* infection. In fact, the World Health Organization (WHO) has recommended two methods based on cellular immune response for latent TB infection (LTBI) (7). The methods have enabled the identification of LTBI and treatment with anti-TB drugs. This is particularly important for stopping the transmission of *Mtb*. In the TB guidelines of the European Union, the interferon-gamma (IFN-γ) release assay (IGRA) has also been suggested as an accessory diagnostic method for active TB (8).

The first cellular immune response-based method is the IGRA, in which peripheral blood mononuclear cells (PBMCs) of an LTBI-suspected individual are exposed to *Mtb* antigens. If the individual has LTBI, memory T cells in the PBMCs will be activated *in vitro* by *Mtb* antigens to release cytokines, including IFN-γ, which can be measured (9). The key reagent for the currently approved IGRA-based latent TB diagnosis is a pool of short peptides derived from two *Mtb* RD1 antigens: early secreted antigen target 6 (ESAT-6) and cell filtrate protein 10 (CFP-10). The advantages of using the ESAT-6 and CFP-10 derived peptide antigens are: 1) distinguish from BCG (bacille Calmette-Guérin) vaccination; 2) stimulate strong cellular immune responses; and 3) overcome MHC restriction as the pooled peptides contain multiple epitopes suitable for different HLA phenotypes. However, quality control in the industrial manufacturing process of multiple peptides requires multiple GMP processes and is labor-intensive and time-consuming. It has posed a limitation to cost-effective manufacturing of diagnostic kits. Consequently, the use of the IGRA assay as a screening test for latent TB for the general population has not become a reality.

Another method of detecting LTBI is the Mantoux skin test based on a purified protein derivative (PPD). This is an *in vivo* method based a delayed-type hypersensitivity (DTH) response in which the antigen presentation of the exogenous PPD antigen leads to a cellular immune response as a consequence of a collective cytokine release. The sign of an immune response is a skin reaction at the PPD injection site. The advantages of this method include the ability to measure cellular immune responses not only based on one cytokine (i.e., IFN-γ) but also on the collective stimulation of other factors (cytokines/chemokines). Furthermore, it a simple and economical test, with no need for *in vitro* cell culture equipment or expensive reagents (tissue culture plates and antibodies); therefore, it is suitable as a screening test in the general population. However, the PPD-based skin test cannot distinguish *Mtb* infection from BCG vaccination.

**Figure 1:**
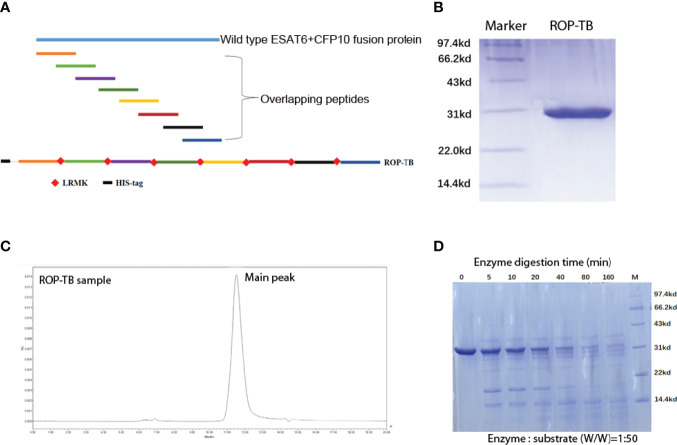
Purification and digestion of ROP-TB. **(A)** Schematic presentation of an ROP-TB antigen. The blue line on top represents the sequence of the fusion protein of ESAT-6 and CFP-10; the short colorful lines are the overlapping peptides covering the full sequence length of ESAT-6-CFP-10. The bottom line represents ROP-TB which is a chain of the overlapping peptides linked by LRMK, the substrate of the enzyme Cathepsin S. **(B)** ROP-TB was expressed by *E. coli* BL-21 and purified using a Ni-NTA affinity column. **(C)** After purification, ROP-TB was analyzed by HPLC to further confirm purity. **(D)** ROP-TB was digested at 37°C by Cathepsin S at 1:50. After digestion, samples were collected at different times and analyzed by SDS-PAGE gel.

The goal of this study is to develop an antigen that can serve as a key reagent for both the IGRA assay and the skin test. The reagent should be able to distinguish *Mtb* infection from BCG vaccination and be suitable for industrial manufacture. We have previously reported using recombinant overlapping peptide (ROP) proteins as stimulants for cellular immunity (10, 11). An ROP is composed of a chained series of overlapping peptide sequences derived from a target protein (e.g., ESAT-6) and covers the whole sequence of the target protein (**Figure 1A**). The overlapping sequences of an ROP are linked by a substrate of protease (e.g., Cathepsin) in the antigen-presenting cells. The ROP can be easily cleaved inside APCs and cross-present to induce potent CD4+ and CD8+ T-cell responses (11). There are several advantages of using ROP: 1) the ease of industrial manufacture, due to only a single GMP processing step and the requirement for one quality control; 2) ROPs are linear sequences containing all possible T cell epitopes which can promiscuously stimulate T cells irrespective of MHC phenotypes; and 3) ROPs do not have tertiary structure, therefore, they do not possess any toxic protein functions of the native microbial/tumor proteins.

Consequently, ROP technology may be a solution to overcome the drawbacks of manufacturing and quality control of the key reagents in the current IGRA assay. In addition, it may also serve as a skin test agent. In this study, our goal was to design and produce an ROP-TB diagnostic agent based on the two *Mtb* antigens, ESAT-6 and CFP-10. We then used *in vitro, ex vivo*, and *in vivo* experiments to demonstrate antigen cross-presentation and induction of cellular immune responses.

## Results

### Design, Expression, Purification, and Enzymatic Digestion of ROP-TB

ROP-TB was derived from ESAT-6 and CFP-10. It was designed to include a sequence of 8 peptides consisting of 35 amino acids per peptide. Each peptide overlapped with its adjacent peptides by 10 amino acids. The eight sequential overlapping peptides covered the complete sequences of ESAT-6 and CFP-10. The cathepsin S substrate sequence (LRMK) was interspersed between each peptide. Cathepsin S is known to exist in professional APCs (10). A schematic presentation of ROP-TB is shown in **Figure 1A**. ROP-TB was expressed by *E. coli* BL21 cells and then purified by Ni NTA affinity chromatography. (**Figure 1B**). The purity of the protein was confirmed by HPLC to be more than 95% (**Figure 1C**).

To remove any potential endotoxins that may have potentially contaminated the product, ROP-TB was further purified by endotoxin removal chromatography. Subsequent endotoxin levels in ROP-TB before application in cell cultures was tested and were <1 EU/mL. To test the effects of endotoxin on the ROP-TB-based IGRA assay, an enzyme-linked immunosorbent spot (ELISPOT) assay study using ROP-TB spiked with endotoxin was carried out. The doses of endotoxin used (up to 33 EU/mL) did not influence the results of the ROP-TB-related ELISPOT (**Supplementary Figure 1**). This is consistent with another report showing that only more than 100 EU/mL of endotoxin induces an ELISPOT response (12).

To test the enzymatic effects of cathepsin S on the protein, ROP-TB was incubated *in vitro* with cathepsin S. After digestion, samples were analyzed using SDS-PAGE. As **Figure 1D** shows, ROP-TB can be digested *in vitro* into peptides of different sizes by cathepsin S (**Figure 1D**).

### Comparing the Metabolic Rate of ROP-TB, TB Peptides, and TB Fusion Protein in APCs

**Figure 2:**
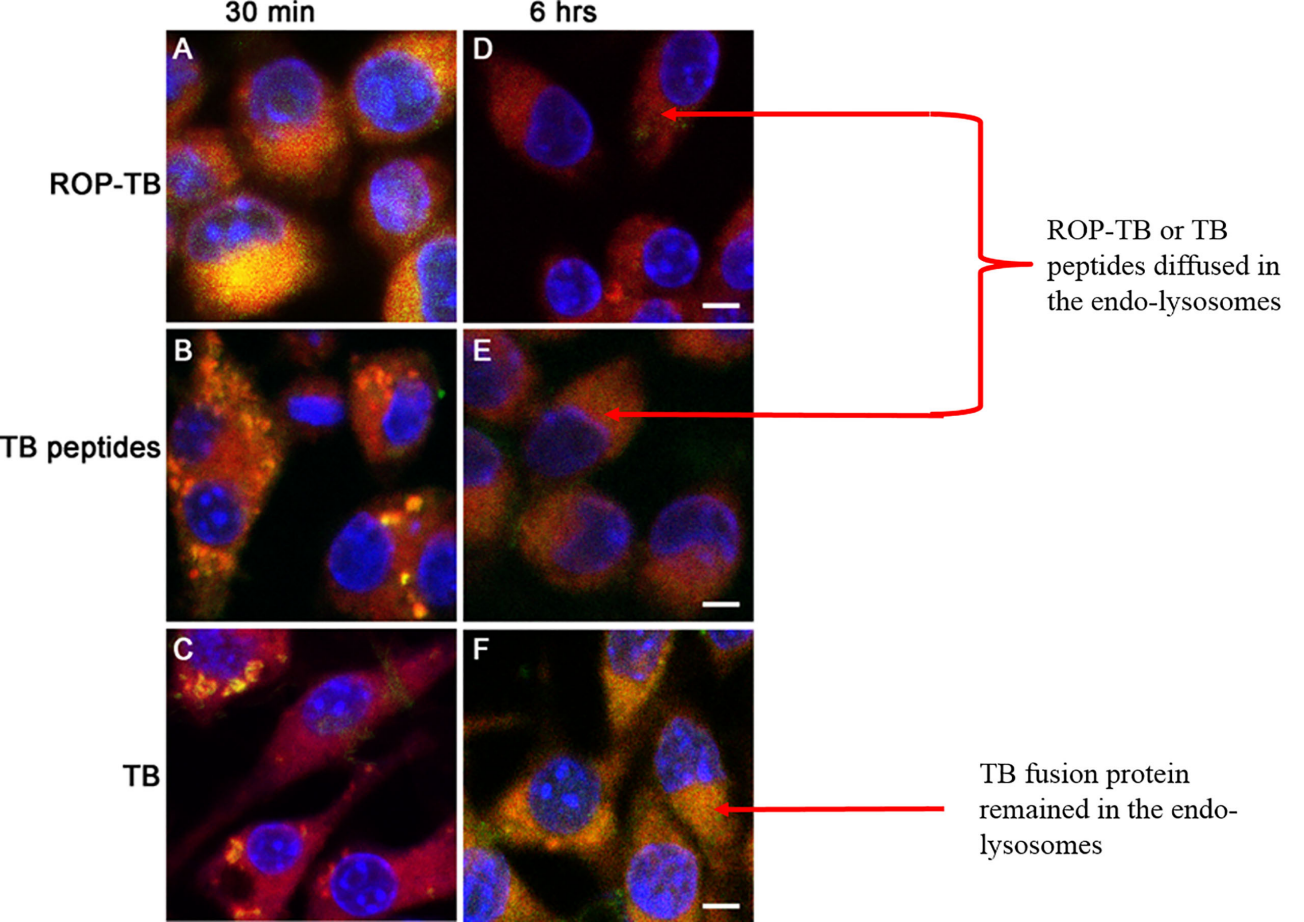
Intracellular localization of ROP-TB, TB peptides, and TB protein in DC2.4 cells. DC2.4 cells were incubated with green fluorophore labeled ROPTB, TB peptides, or TB protein for 30 min and 6 hours followed by confocal microscopy analysis (63x objective). DC2.4 cells were co-stained with lysotracker red for visualizing endo-lysosomes. The nucleus was counterstained with DAPI. **(A–C)** ROP-TB, TB peptides, and TB fusion protein are internalized by DC2.4 within 30 min of incubation and are largely co-localized within endolysosomes compartment. **(D, E)** ROP-TB and TB peptides diffused in the endo-lysosomes after 6 hours. **(F)** TB fusion protein remained in the endo-lysosomes after 6 hours. Scale bar: 10 µm.

To understand the cross-presentation efficiency of ROP-TB in APCs, it is important to investigate its metabolic rate in the endolysosome. We, therefore, compared ROP-TB with TB peptides and TB wild-type protein (fusion of ESAT-6 and CFP-10) by tracing their intracellular localization at different time points in the mouse dendritic cell line DC2.4 cells. ROP-TB, TB peptides, and TB protein were labeled with a green fluorescent dye (WV=530/30nm) and were incubated with DC2.4 cells for 30 min and 6 h. DC2.4 cells were co-incubated with Lysotracker to emit red fluorescence (WV=630/30nm). The uptake of ROP-TB, TB peptides, or TB protein is shown in green, and endolysosomes are shown in red under a confocal microscope. The co-localization between red and green colors represents the approximate location of the two objects. As shown in **Figures 2A–C**, ROP-TB, TB peptides, and TB fusion protein are internalized by DC2.4 within 30 min of incubation and are largely co-localized within endolysosomes. This indicates that these antigens are located within the endolysosomal compartment. After 6 hours, the green fluorescence in **Figures 2D, E** (ROP-TB and TB peptides) but not in **Figure 2F** (TB fusion protein), disappeared (**Figures 2D–F**), suggesting that ROP-TB and TB peptides have been processed more rapidly (**Figure 2**).

### Development of T Cell-Based Diagnosis in *Mtb-*Infected Mouse Model: ROP-TB and Pooled Synthetic TB Peptides Induce Specific T Cell Responses

**Figure 3:**
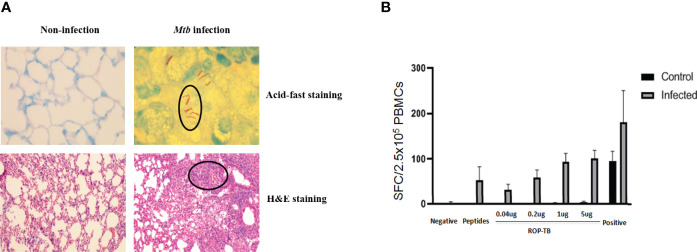
ROP-TB stimulates specific T cell immune response in the *Mtb-*infected mouse model **(A)** Establish *Mtb-*infected mouse model by intranasal infection of *Mtb*. Upper panels: acid-fast staining of lung alveolar in noninfected (left) and infected (right) mice. *Mtb* are observed in the lung (circled) only in infected mice (right). Lower panels: H&E staining of histopathological changes of the lungs in non-infected mice (left) and infected mice (right). Marked lymphocyte infiltration in the interstitial and alveolar space is present only in infected mice (right) together with pulmonary edema, alveolar distortion, and thickening of the alveolar wall. **(B)** Specific T cell responses of infected mice. Responses of spleen cells from noninfected (control) and infected mice to different doses of ROP-TB are only seen in infected mice. Negative control: culture medium (R10); stimulant: peptides (5 μg/well); ROP-TB with 4 different doses (0.04, 0.2, 1 and 5 μg/well); Positive control: PHA (5 μg/well).

To test whether ROP-TB can be developed as a diagnostic agent for T cell function, we assessed specific T cell responses induced by synthetic ROP-TB or *Mtb* peptides *ex vivo* in a mouse model. We first established the test model by infecting BALB/c mice with *Mtb* (H37R1) intranasally (**Figure 3**). Infectivity was validated by acid-fast staining and Hemotoxin-Eosin (H&E) staining of lung tissue (**Figure 3A** upper panel). The lungs of infected mice clearly showed infection with H37R1 and presented lesions when compared to non-infected mice (**Figure 3A**, lower panel).

Spleen cells from *Mtb-infected* mice were then isolated and stimulated with different concentrations of synthetic ROP-TB and *Mtb* peptides. T cell responses to these antigens were measured by the IGRA assay (ELISPOT). As shown in **Figure 3B**, ROP-TB stimulates stronger specific T cell responses than synthetic peptides, indicating that ROP-TB is a more sensitive antigen in the mouse model than synthetic peptides in detecting T cell responses.

### Delayed-Type Hypersensitivity Responses to ROP-TB, ESAT-6/CFP-10 and PPD in Guinea Pigs Infected With Mtb and Cattle Infected With *M. bovis*

**Figure 4:**
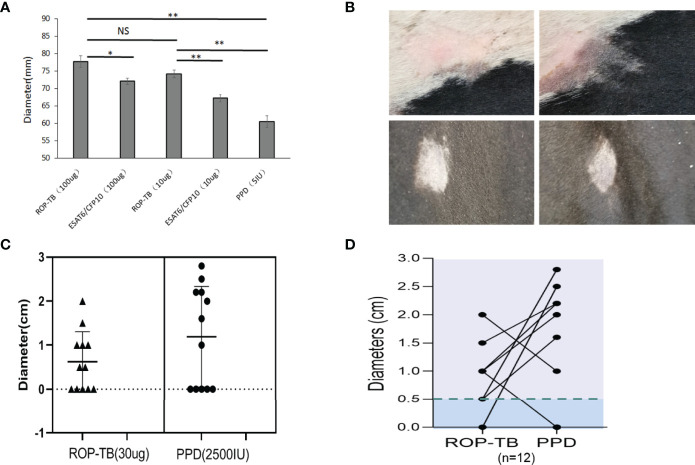
Delayed-type hypersensitivity to ROP-TB, ESAT-6/CFP10, and PPD in *Mtb* infected guinea pigs and *M. bovis*-infected cattle. **(A)** Skin reaction in guinea pigs: the bars show the size of skin reaction to two doses (100 μg and 10 μg) of ROP-TB and ESAT-6/CFP-10, respectively in guinea pigs (n=4 per group). PPD at 5 IU was used as a comparison. Delayed-type hypersensitivity to ROP-TB and PPD in cattle infected with tuberculosis (n=12). **(B)** Skin reaction to cattle: Representative positive skin reactions to ROP-TB (upper left panel) and PPD (upper right panel); and negative reactions to ROP-TB (lower left panel) and PPD (lower right panel). **(C)** The size of the skin reactions of all 12 cattle to ROP-TB (left panel) and PPD (right panel). **(D)** The relationship of DTH of each head of cattle to ROP-TB and PPD. “*” (P<0.05): significant difference, “**” (P<0.01): extremely significant difference. NS; no statistically significant difference.

Delayed-type hypersensitivity (DTH) reflects T cell responses to antigen stimulation *in vivo*. To investigate whether ROP-TB can be processed and presented *in vivo* to specific T cells, guinea pigs were infected with *Mtb* H37R1, and DTH-related skin reactions to ROP-TB, ESAT-6/CFP-10, and PPD (purified protein derivative) were investigated. Both ROP-TB and ESAT-6/CFP-10 trigger positive responses to DTH, as well as PPD (**Figure 4A**). DTH elicited by ROP-TB was stronger than that elicited by ESAT-6/CFP-10 and PPD in 100 μg and 10 μg doses (p<0.01) (**Figure 4A**).

Bovine TB (*M. Bovis*) epidemics have been reported in many parts of the world. This has posed a risk for humans drinking infected, unpasteurized milk. Practically all cattle on a farm need to be slaughtered even if only one head of cattle is found to be infected with *M. bovis*. This causes great economic loss. Thus, as a preventative measure, a tuberculin-based skin test using a PPD is usually performed to screen for infected cattle (13). BCG may also be used as a vaccine for *M. bovis* infection (14) if there is another available diagnostic reagent which will not be interfered by the vaccination.

Since *M. bovis* and *Mtb* share the same ESAT-6 and CFP-10 sequences (15), we wondered whether our ROP-TB reagent could be used as another choice of *M. bovis* skin test reagent. Thus, the application ROP-TB as a diagnostic reagent for *M. bovis* infection was tested in cattle from two farms (Farm 1, n=12 and Farm 2, n=40, respectively) recruited for ROP-TB and PPD- based skin tests. As shown in **Figure 4B**, both ROP-TB and PPD stimulates skin reactions in the cattle from Farm 1. Among the 12 cattle, 7 show positive skin reactions to ROP-TB stimulation (**Figure 4C**, left panel) and 7 to PPD stimulation (**Figure 4C**, right panel). **Figure 4D** shows the interrelationship of ROP-TB-stimulated and PPD-stimulated skin reactions in these cattle. None of the cattle in Farm 2 produced a positive skin reaction to antigens. **Table 1** summarizes the skin reactions of both farms. The sensitivity and specificity of ROP-TB with PPD are 85.7% and 97.7%, respectively (**Table 1**).

**Table 1:** Delayed-type hypersensitivity skin test of ROP-TB and PPD cattle.

Antigen	Farm 1 N=12		Farm 2 N=40	Sensitivity (%)^a^	Specificity (%)^b^
	Positive	Negative	positive		
PPD	7	5	0	85.7	97.7
ROP-TB	6	6	0

a Percentage of positive responders with ROP-TB in positive responders with PPD.

b Percentage of negative responders with ROP-TB in positive responders with PPD.

### ROP-TB Overcomes HLA Restriction in Stimulating T Cells in TB Patients

To observe whether ROP-TB can promiscuously stimulate the memory T cell response in humans, we tested ROP-TB for a specific T cell immune response measured by ELISPOT in a population of diverse HLA phenotypes. We recruited 18 patients with tuberculosis who were diagnosed by sputum test, acid-fast staining, and lung computed tomography (CT) scan. The demographic data for these patients are summarized in **Table 2**. Their HLA phenotypes are shown in **Table 3**. We then compared the ELISPOT response of the PBMC of the 18 patients with the following antigens: 8 individual overlapping synthetic peptides derived from ESAT-6 and CFP-10, pooled peptides of the 8 overlapping synthetic peptides derived from ESAT-6 and CFP-10, and ROP-TB (which is composed of linked and chained peptides of the 8 peptide sequences). As shown in **Table 4**, the 18 patients having different HLA types (**Table 3**) respond to different individual peptides. However, most patients, 16/18 in ROP-TB and 15/18 in pooled peptides, respond to the pooled peptides and ROP-TB (**Table 4**). This experiment shows that ROP-TB, like the pooled peptides, is able to stimulate memory T cell responses in different HLA types of TB patients.

**Table 2:** Clinical data of the 18 *Mtb* patients.

Patient	Sex	Age	White blood cell count (10^9^/L)	Tuberculin Test	Acid-fast staining	X-pert	Sputum culture	CT
WS-118	Female	31	5.03	++	—	+	—	Plaque shadows in the left lung
WS-119	Female	12	4.4	++	—	+	—	Plaque shadows in both lungs,
WS-120	Female	40	4.28	++	+	+		Plaque shadows in the right lower middle lung
WS-121	Male	77	9.29	+	+	—		Multiple cavities in both lungs
WS-127	Male	75	8.18	+	+		+	Both lung lesions; damage to the left lung
WS-129	Male	63	2.76	++	+	+	—	Partial damage to the right lung
WS-130	Male	39	5.34	++	+	+	—	Plaque shadows in the left lung
WS-139	Male	30	8.56	++	+	+	—	Multiple high-density shadows in both lungs
WS-140	Male	64	4.54	++	—	+	—	Nodules in the upper lobe of the right lung with a few surrounding patchy shadows
WS-141	Female	22	4.45	++	—	—	—	Patchy shadows in the upper right lobe of the lung
WS-142	Female	39	7.08	++	—	+		Patchy shadows in the upper lobe of the left lung,
WS-143	Female	48	2.49	+	—			Plaque shadows and pleural effusion in the left lung
WS-157	Female	27	5.99	++	—			High-density patchy shadows in both lungs
WS-158	Female	77	5.3	++	–			Density patchy shadows in both lungs
WS-159	Male	58	7.96	+		+		Multiple cavities in both lungs
WS-160	Female	51	7	++	—	—	—	Density patchy shadows in both lung
WS-161	Male	59	3.58	+	+	+		Density patchy shadows in both lungs; pleural effusion in the left lung
WS-162	Female	72	6.23	++	+			Plaque, shadow/cavities in the left lung; plaque shadows in the right lung

“++” strong positive, “+”positive, “—”negative.

**Table 3:** HLA phenotypes of the 18 *Mtb* patients.

Patient	HLA-A	HLA-B	HLA-C	HLA-DRB1	HLA-DQB1
WS-118	A*11:02 A*33:03	B*40:01 B*58:01	C*03:02 C*07:02	DRB1*03:01 DRB1*08:03	DQB1*02:01 DQB1*06:01
WS-119	A*24:08 A*24:02	B*15:01 B*54:01	C*01:02 C*01:02	DRB1*11:01 DRB1*12:01	DQB1*03:01 DQB1*03:01
WS-120	A*02:07 A*24:02	B*46:01 B*52:01	C*01:02 C*07:02	DRB1*08:03 DRB1*14:10	DQB1*05:03 DQB1*06:01
WS-121	A*02:07 A*11:01	B*13:01 B*54:01	C*01:02 C*07:02	DRB1*04:05 DRB1*12:02	DQB1*03:01 DQB1*04:01
WS-127	A*24:02 A*31:01	B*46:01 B*55:02	C*01:02 C*01:02	DRB1*14:05 DRB1*14:01	DQB1*05:02 DQB1*05:03
WS-129	A*02:10 A*30:01	B*13:02 B*40:06	C*06:02 C*08:01	DRB1*07:01 DRB1*12:01	DQB1*02:03 DQB1*03:04
WS-130	A*02:03 A*02:01	B*15:02 B*35:01	C*03:03 C*08:01	DRB1*12:02 DRB1*15:01	DQB1*03:01 DQB1*06:02
WS-139	A*30:01 A*33:03	B*13:02 B*58:01	C*03:02 C*06:02	DRB1*07:01 DRB1*13:02	DQB1*02:02 DQB1*06:09
WS-140	A*11:01 A*24:02	B*15:01 B*35:01	C*03:03 C*04:01	DRB1*09:01 DRB1*15:01	DQB1*03:03 DQB1*06:02
WS-141	A*02:07 A*11:01	B*15:58 B*38:02	C*01:02 C*07:02	DRB1*04:03 DRB1*09:01	DQB1*03:02 DQB1*03:03
WS-142	A*11:01 A*33:03	B*15:02 B*58:01	C*03:02 C*08:01	DRB1*12:02 DRB1*13:02	DQB1*03:01 DQB1*06:09
WS-143	A*11:01 A*24:02	B*15:27 B*35:01	C*03:03 C*04:01	DRB1*11:01 DRB1*15:01	DQB1*03:01 DQB1*06:02
WS-157	A*02:01 A*31:01	B*15:11 B*15:01	C*01:02 C*03:03	DRB1*07:01 DRB1*09:01	DQB1*02:02 DQB1*03:03
WS-158	A*24:02 A*24:02	B*15:27 B*40:02	C*03:04 C*04:01	DRB1*04:06 DRB1*09:01	DQB1*03:02 DQB1*03:03
WS-159	A*11:01 A*24:02	B*40:06 B*40:01	C*07:02 C*08:01	DRB1*08:03 DRB1*09:01	DQB1*03:03 DQB1*06:01
WS-160	A*03:01 A*33:03	B*35:01 B*52:01	C*07:02 C*12:02	DRB1*15:01 DRB1*15:02	DQB1*06:01 DQB1*06:02
WS-161	A*11:02 A*11:01	B*15:02 B*27:04	C*08:01 C*12:02	DRB1*08:03 DRB1*12:02	DQB1*03:01 DQB1*06:01
WS-162	A*11:01 A*11:02	B*15:02 B*40:01	C*07:02 C*08:01	DRB1*09:01 DRB1*12:02	DQB1*03:01 DQB1*03:03

**Table 4:** ELISPOT responses of the 18 *Mtb* patients to various forms of TB antigens.

Patient	P1	P2	P3	P4	P5	P6	P7	P8	Pool of P1-P8	ROP	CFP-10	ESAT-6
WS-118	—	—	—	+	—	—	+	+	+	+	+	+
WS-119	—	+	—	—	+	—	+	+	+	+	+	+
WS-120	+	—	+	+	+	+	+	+	+	+	+	+
WS-121	—	—	—	—	+	—	+	—	+	+	+	+
WS-127	—	—	—	—	—	—	—	—	—	—	—	—
WS-129	—	—	—	—	—	—	—	+	—	—	—	+
WS-130	—	—	+	—	—	—	—	—	+	+	+	+
WS-139	—	—	—	—	—	—	+	—	—	+	—	+
WS-140	—	—	+	—	—	—	+	—	+	+	+	+
WS-141	—	+	+	+	—	—	+	+	+	+	+	+
WS-142	—	—	+	—	+	—	+	+	+	+	+	+
WS-143	—	—	—	—	—	—	—	+	+	+	—	+
WS-157	—	—	—	—	+	—	—	—	+	+	+	
WS-158	+	+	+	+	—	—	—	—	+	+	+	—
WS-159	+	—	—	—	+	—	—	—	+	+	+	—
WS-160	+	—	+	—	—	—	+	+	+	+	+	+
WS-161	—	—	+	+	+	+	—	—	+	+	+	+
WS-162	+	—	+	—	—	—	—	—	+	+	+	—

“+” Represent positive, “—” Represent negative.

## Discussion

Monitoring and detecting specific T cell responses is important for many diseases, such as tumors, infectious diseases, and inflammatory diseases. However, currently, TB is the only disease indication for commercially available, T cell-based diagnostic assays, which consist of a skin test and IGRA assays. The difficulties of developing a specific T-cell-based test include the identification of specific antigens that can promiscuously stimulate T cells from various MHC backgrounds; the manufacture of such antigens without labor intensive processes such as multiple GMP manufacturers and/or quality controls; and the requirement of cell culture facilities for their execution, which may hamper the use of the assays in a hospital setting, especially in remote areas in developing countries.

For the two TB diagnostic methods currently used clinically, the key reagents/antigens are derived from different origins. The antigen used in the skin test assay is a PPD, while the antigen used in the IGRA assays consists of a peptide pool derived from ESAT-6 and CFP-10 (16, 17). PPD does not distinguish TB infection from BCG vaccination. Although, pooled ESAT-6/CFP-10 peptides can distinguish TB infection from BCG vaccination; they may not be suitable for industrial manufacture due to complicated multiple quality controls and regulation issues, especially in the case of skin tests in which the antigen will be injected into a human body.

The aim of this study was to develop an antigen suitable for both IGRA and a diagnostic skin test. We designed a chain of overlapping peptides (ROP) linked by the enzyme substrate cathepsin S. This antigen is cleavable in APCs where the enzyme cathepsin S is located. After degradation by the enzyme, the antigen will undergo presentation and/or cross-presentation to stimulate T cells. Our data from this study showed that ROP-TB could be produced and purified recombinantly using the *E. coli* system (**Figure 1**). The ROP-TB can be cross presented in APC (**Figure 2**) to stimulate T cells (**Figure 3**) in mice. It has also been shown to induce skin reactions in guinea pigs and cattle (**Figure 4**). Finally, ROP-TB promiscuously stimulates human T cell responses (**Table 4**).

There are several advantages of this artificial ROP antigen. First, ROP-TB is derived from ESAT-6/CFP-10 which is different from BCG and hence it is specific for TB infection but not for BCG vaccination. Second, since the antigen is produced by *E. coli*, which can be adapted to large-scale manufacturing processes, the costs are reasonably low. Moreover, ROP-TB is produced as one entity; therefore, only one quality control step is required in the downstream process of manufacture. Furthermore, unlike pooled peptides, ROP-TB is convenient (as it is applied as a single substrate), which is useful when applying for regulatory approval. Third, like pooled peptides, the antigen is promiscuous and stimulates T cells from individuals of different HLA phenotypes. This has been demonstrated by this study (**Tables 3**, **4**). Fourth, ROP-TB does not have share the same conformation structure as native antigens (ESAT-6/CFP-10), therefore, it lacks the potential pathogenic functions of native ESAT-6/CFP-10. This is especially important if it is intended to be used as a skin test reagent. Fifth, ROP-TB can also be produced under other forms such as DNA, mRNA, and viral/bacterial vectors, which represents an added advantage to using ROP-based antigens.

In summary, we have demonstrated that ROP-TB can be used as a new diagnostic platform technology for tuberculosis diagnostics in both IGRA assays and skin tests.

## Methods

### Molecular Cloning, Protein Expression, and Purification

The cDNA genes encoding native ESAT-6-CFP-10 and the ROP-TB respectively were designed and synthesized as previously described (6).

For purification, the corresponding plasmids were transformed into *E. coil* BL21 (Solarbio, China). A single colony was harvested in LB medium (50 μg/mL Kanamycin) and cultured overnight. Bacteria from the overnight culture were then inoculated into the new LB medium at a 1:100 ratio and cultured until the OD600 reached 0.6. Isopropyl β- d-1-thiogalactopyranoside (IPTG, Solarbio, China) was added at a final concentration of 0.5 mM and the bacteria were collected by centrifugation after 16 h of incubation. Lysis buffer (20mM Tris-HCl, 0.5 M NaCl, 2 mM EDTA, pH 8.0) was used to resuspend the bacteria, which were then lysed by sonication. The insoluble fractions were separated by centrifugation at 15,000 g for 1 hour.

The protein was further purified by nickel affinity chromatography using Ni-NTA (GE, USA). To detect expressed soluble TB protein (ESAT-6-CFP-10), Ni-NTA resin was added to the soluble fractions for 1 h. The protein was washed with lysis buffer containing 10 mM imidazole (washing buffer) and eluted with lysis buffer containing 300 mM imidazole (elution buffer). The insoluble ROP-TB in the pellet was resuspended in 8 M urea buffer and centrifuged at 15,000 g for 1 h. The rest procedures were similar to those of ESAT-6-CFP-10 except that the washing and elution buffer contained 8 M urea. For refolding, the eluted proteins (ESAT-6-CFP-10 and ROP-TB) were exchanged by PBS for dialysis.

### Endotoxin Removal and Limulus Amebocyte Lysate (LAL) Assay for Endotoxin

Ni-NTA purified ROP-TB was further purified by endotoxin removal chromatography (BIOENDO, China). Briefly, the column (1.5 mL) was washed with balanced buffer, then ROP-TB was flowed through the column at 0.5 mL/min and the effluent collected.

The endotoxin present in ROP-TB was detected using the Limulus amebocyte lysate assay (BIOENDO, China) following the manufacturer’s instructions. Briefly, ROP-TB was diluted with endotoxin-free water by serial two-fold dilutions, then diluted ROP-TB was added, respectively, to the Limulus amebocyte lysates, which were dissolved in 100 μL endotoxin-free water. The mixtures were incubated in ampoule bottles at 37°C for 1 hour. If a gelatinous substance formed at a certain dilution, it was considered positive: Endotoxin concentration was calculated as maximum positive × 0.125 EU/mL, which indicated the sensitivity of Limulus amebocyte lysate.

### Antigen Digestion *In Vitro* and Trafficking in DCs by Confocal Microscopy

Purified protein (ROP-TB) in PBS and 4mM DTT was incubated with Cathepsin S (SinoBiological, China) at 37°C for 0.5, 1, 2, 4, 8, and 22 h. The mass ratios of enzyme and protein in digestion were 1:50 and 1:100, respectively. Samples with different digestion times were run through SDS-PAGE for measuring enzyme activity.

To observe antigen uptake, 1 μM ROP-TB, TB peptides, and TB protein (ESAT-6-CFP-10) were incubated with DC2.4 cells (a gift from K Rock of Massachusetts University, United States) in culture plates (3x10^5^ per dish) at 37°C for 30 minutes or 6 hours. ROP-TB, TB peptides, and TB protein marked with the LIVE/DEAD cell stain kit (Invitrogen, USA). To stain endolysosomes, 10 μL of 1:200 diluted (red) Lysotracker (Invitrogen, USA) was added to the cell culture one hour before incubation. After incubation, cells were washed and fixed with 4% paraformaldehyde for 10 min. The samples were further stained with DAPI (Solarbio, USA) prior to observation under a confocal microscope (ZEISS, LSM800).

### *In Vitro* of IGRA-Based T Cell Diagnosis in Mice

The mouse experiment was outsourced to the Shanghai Public Health Center (Shanghai, China). All procedures were approved by the Committee of the Shanghai Public Health Center and followed the national guidelines for the use of experimental animals. Briefly, all mice (BALA/c, age 6–8 weeks, SPF) were infected intranasally with *Mtb* (H37R1) at 5x10^5^ CFU in 40 μL. After infection, the animals were housed in cages kept in laminar flow safety enclosures in a Level III biosafety facility.

To verify the establishment of the *Mtb* model, the lung tissues of infected and uninfected mice were sectioned for fast acid staining. The sections were placed in a staining dish and stained with Carbol Fuschin. After heating to steam and incubation for 5 min, the lung sections were washed with slow running water, decolorized with acid alcohol, counterstained with Malachite green for 1 min, and then air-dried. The final sections were positive when red and negative otherwise. The procedure of H&E staining (Sigma)for the evaluation of lymphocyte infiltration is the following: samples were first fixed overnight with 4% paraformaldehyde. After embedding in paraffin, the samples were cut into 2-μm thick sections and subjected to H&E staining. Histopathological analysis was performed using a Zeiss microscope.

The IGRA-based T cell function assay was performed using ELISpot kits (Mebtech, Sweden) (6). Briefly, spleen cells were separated and incubated at 2.5x10^5^ cells/well with 5 μg/well ROP-TB or peptides at 37°C for 24 h. Then, 96-well culture plates were precoated with IFN-γ antibodies. After the incubation, the cells were discarded, and the plates were washed with PBS followed by incubation with biotinylated anti-IFN-γ antibodies for 2 h at room temperature. The plates were washed with PBS and an enzyme-labeled anti-biotin antibody was added for 1 h. Finally, BCIP/NBT was used for color development, and the reaction was stopped by washing plates with tap water. Spots were counted with an Elispot reader (Autoimmun Diagnostike, Strasburg, Germany).

### *Ex Vivo* IGRA-Based T Cell Diagnosis in Human

The human experiments were reviewed and approved by the Ethics Committee of Changzhou No. 3 People’s Hospital. Eighteen patients with tuberculosis infection were recruited from the hospital and provided informed written consent prior to the collection of their detailed clinical data (**Table 2**). HLA typing of the patients was performed by Weihe Biotechnology INC (Jiangsu, China).

IGRA-based T cell testing was performed using ELISpot kits (Mebtech, Sweden). Briefly, 2.5x10^5^/150 μL of PBMCs were stimulated overnight with 5 μg/well of ROP-TB, either pooled or as singular peptides in anti-IFN-γ-Ab precoated plates (Millipore, Bedford, MA). Cells were discarded and biotinylated anti-IFN-γ antibodies were added for a 2-h incubation at room temperature followed by another 1-h incubation at room temperature with an enzyme-labeled anti-biotin antibody. After the color development, the reaction was stopped by washing the plates with tap water, and the plates were air dried. Spots were counted with an Elispot reader (Autoimmun Diagnostike, Strasburg, Germany).

### *In Vivo* Diagnosis of Tuberculosis (Skin Test) in Guinea Pigs

The experiment was outsourced to the Shanghai Public Health Center (Shanghai, China). All animals were infected with *Mtb* (H37R1) of 0.5 mL (5x10^4^ CFU) through nasal drop. After infection, the animals were housed in cages contained within laminar flow safety enclosures in a Level III biosecurity facility.

The DTH-based skin test was performed as follows: guinea pigs were divided into six groups of five animals per group. They were injected intradermally on the flank with 0.1 mL of ESAT-6-CFP-10 (1 mg/mL, 0.1 mg/mL), ROP-TB (1 mg/mL, 0.1 mg/mL), national standard and negative control (physiological saline). The diameters of the erythema were read after 24–72 hours.

### *In Vivo* Skin Test of TB in Cattle

The experiments were approved by the Ethics Committee of Southwest University (Ethics approval number: IACUC-20210320-03). Cattle from two farms (n=12 and n=40, respectively) were recruited. To evaluate the potential of a DTH-based skin test with ROP-TB, we selected commercially available PPD, which is the standard reagent for the diagnosis of *M. bovis* TB, to be compared with ROP-TB in the experiments. The PPD skin test was performed following the standard Chinese diagnostic technique for tuberculosis in animals. ROP-TB antigen (30 μg/animal) was injected into a volume of 0.1 mL on one side of the cow neck, while PPD (2500 IU/animal) on the other side. Skin thicknesses at injection sites were measured between 48 and 72 h of injection. For the readout of the skin test, if the skin thickness of the cattle was ≥ 4 mm, the animal was considered skin test-positive and negative if the thickness was <2 mm. In the case that the difference was nearly or equal to 2 mm, the result was considered inconclusive.

### Statistical Analysis

Statistical analysis was performed with Student’s t test. P values <0.05 were considered statistically significant. **Figure 4A** was generated and analyzed using GraphPad Prism version 8 (GraphPad Software Inc.).

## Data Availability Statement

The original contributions presented in the study are included in the article/**Supplementary Material**. Further inquiries can be directed to the corresponding authors.

## Ethics Statement

The human experiments were reviewed and approved by the Ethics Committee of Changzhou No 3 People’s Hospital. The patients/participants provided their written informed consent to participate in this study. The mouse and guinea pig experiments were approved by the Committee of Shanghai Public Health Centre and the cattle experiments were approved by Ethic Committee of Southwest University. Written informed consent was obtained from the owners for the participation of their animals in this study.

## Author Contributions

SJ, WL, and JW designed the study and wrote the manuscript; QZ performed most of the experiments and carried out data analysis; XL performed the cow skin test and ELISPOT assays; ST helped to carry out the HLA analysis; DC performed the purification of ROP-TB; YG and LG helped to collect patient samples; RZ participated in the experimental design and management. All authors contributed to the article and approved the submitted version.

## Funding

The authors declare that this study received partial funding from Oxford Vacmedix UK Ltd and CBI. The funders were not involved in the study design, collection, analysis, interpretation of data, the writing of this article, or the decision to submit it for publication. This research was also supported by National Key Research and Development Program of China (2018YFE0192500) and the Key Research and Development Program of Changzhou (CE20135023, CE20185016). SJ is funded by Innovate UK.

## Conflict of Interest

QZ, XL, YG, DC, RZ, WL and SJ are employees and/or shareholders of Oxford Vacmedix (Changzhou) Ltd which develops TB diagnostic kits. WL is employed by Shanghai JW Inflinhix Ltd (a subsidy company of Oxford Vacmedix(Changzhou)).

The remaining authors declare that the research was conducted in the absence of any commercial or financial relationships that could be construed as a potential conflict of interest.

## Publisher’s Note

All claims expressed in this article are solely those of the authors and do not necessarily represent those of their affiliated organizations, or those of the publisher, the editors and the reviewers. Any product that may be evaluated in this article, or claim that may be made by its manufacturer, is not guaranteed or endorsed by the publisher.

## Supplementary Material

Supplementary Figure 1 ELISPOT responses to ROP-TB spiked with different concentrations of endotoxin in two *Mtb-*infected patients and two healthy donors. Negative control: culture medium (R10); stimulant: ROP-TB (5 μg/well); endotoxin with 3 different doses (33, 6.6 and 3.3 EU/mL); Positive control: PHA (5 μg/well).
