# Supplementary figures and images for: In Vitro and In Vivo Antigen Presentation and Diagnosis Development of Recombinant Overlapping Peptides Corresponding to Mtb ESAT-6/CFP-10
Source: Front Immunol. 2022 Jun 16;13:872676. doi: 10.3389/fimmu.2022.872676 (PMC9246674; doi:10.3389/fimmu.2022.872676)

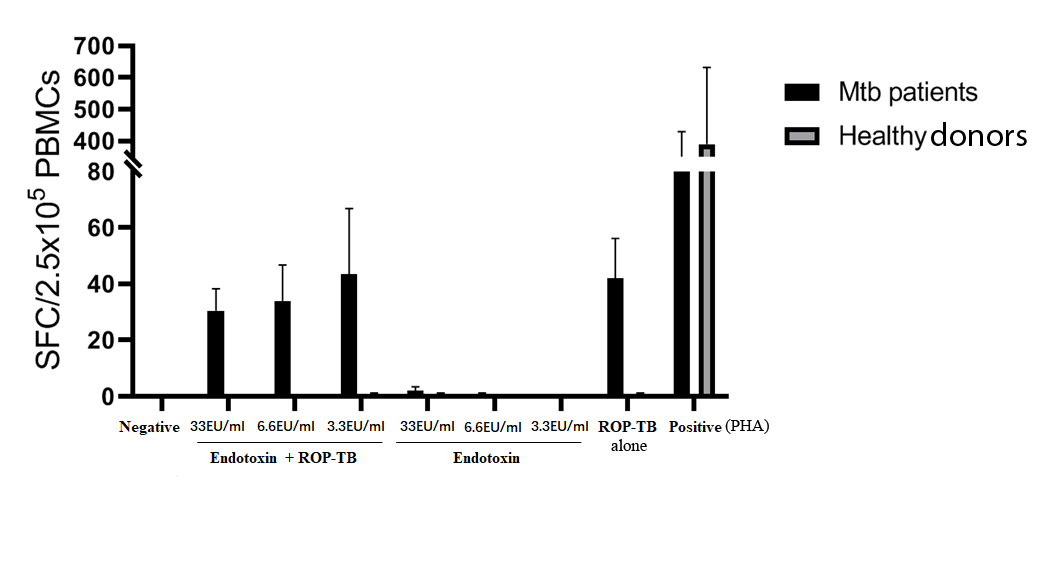

Supplement: Supplementary Figure 1 — ELISPOT responses to ROP-TB spiked with different concentrations of endotoxin in two Mtb-infected patients and two healthy donors. Negative control: culture medium (R10); stimulant: ROP-TB (5 μg/well); endotoxin with 3 different doses (33, 6.6 and 3.3 EU/mL); Positive control: PHA (5 μg/well). [file Image_1.tif]
